# Analysis of association of bandage contact lens with serious vision-threatening diseases and their management

**DOI:** 10.1186/s12886-024-03632-1

**Published:** 2024-08-23

**Authors:** Helena Siegel, Daniel Böhringer, Kilian Rhein, Anne-Marie Shirley Kladny, Thomas Reinhard

**Affiliations:** Eye Center, Medical Center – University of Freiburg, Faculty of Medicine, University of Freiburg, Killianstraße 5, 79106 Freiburg im Breisgau, Germany

**Keywords:** Bandage contact lens, Safety, Adverse events, Endophthalmitis

## Abstract

**Background:**

Bandage contact lenses are important aids for aftercare following ocular surgery and for a wide variety of ocular surface conditions. However, bandage contact lenses also bear the risk of fostering microbial infections of the cornea. We herein report the safety profile of bandage contact lenses from a comprehensive review of medical records in a tertiary care eye hospital in Germany.

**Methods:**

We identified 638 consecutive patients who had been prescribed at least one bandage lens during the past 10 years. For these, we performed a computerized search for the following adverse events: (1) endophthalmitis, (2) emergency keratoplasty and (3) vision loss of at least 2 lines according to the Early Treatment Diabetic Retinopathy Study (ETDRS). We manually assessed the relatedness of each event to the bandage lens. Events later than 100 days following the bandage lens prescription were not considered to be related to bandage contact lenses.

**Results:**

We observed 267 adverse events, with 120 occurring within 100 days after bandage lens prescription. This left a total of 18 endophthalmitis events, 21 penetrating keratoplasties and 81 eyes with vision loss of at least 2 ETDRS lines (for individual review of relatedness). Only two episodes of endophthalmitis could be linked to bandage lens wear. All other adverse events were due to causative conditions that had already been present prior to bandage contact lens insertion.

**Conclusions:**

Severe adverse events after bandage contact lens wear are not uncommon because lenses are used in patients suffering from preexisting ocular conditions. However, severe adverse events were almost never caused by the bandage contact lenses directly in our hands. We therefore conclude that bandage contact lenses are safe given proper ophthalmological supervision.

## Background

Hydrogel and silicone-hydrogel contact lenses are important clinical aids to support epithelial healing in chronic ocular surface diseases such as neurotrophic keratitis [1–5]. The term bandage lens has been coined for all kinds of soft contact lenses when used in this intent. However, very few hydrogel and silicone-hydrogel contact lenses have been specifically labeled for this indication [6].

The major mode of action is to shield the fragile epithelium from the abrasive effects of blinking and from other mechanical hazards to the ocular surface. This shielding promotes epithelial migration and protects Bowman’s membrane and thereby minimizes the risk of haze formation. Further modes of action include prevention of epithelial damage of the ocular surface in areas desiccated from permanent exposure due to eyelid disorders.

Another mainstay of bandage lenses is aftercare in ocular surgery, mostly to alleviate pain from corneal abrasions or to provide tectonic support, e.g., in cases of hyperfiltrating trabeculectomies and to support epithelial regeneration after procedures such as corneal crosslinking, laser-assisted in situ keratomileusis, phototherapeutic keratectomy, and superficial keratectomy for Salzmann’s nodular degeneration. In most cases, soft bandage contact lenses (BCL) are used for a defined period of time until the healing process is completed [7]. However, bandage lenses can also be used for longer periods, e.g., to protect the cornea from eyelid disorders to bridge the delay until definitive surgical repair is feasible.

Different lens types are usually used for this application. However, hydrogel lenses (SiHy) are predominantly used. Either conventional diameters (approximately 14 mm) or large diameters (14.5 up to 22.5 mm) are available, with oxygen permeability (Dk) decreasing with increasing lens size.

The usage of bandage contact lenses has to be weighed against potential risks and side effects. Regarding oxygen permeability, large diameter soft hydrogel and SiHy contact lenses usually meet at least the criteria set forth by Holden and Mertz of DK/t 34.3 × 10^− 9^ to allow the cornea to return to normal thickness soon after eye opening following sleep as a compromise criteria for acceptability for extended wear [8]. Following the goal of avoiding any anoxia while taking into account the pH effect and tear film layer, the contact lens Dk/t for delivering enough oxygen to basal epithelial cells has been determined to be 23 for open eyes and 89 for closed eyes. To avoid any anoxia even in the total stroma, the Dk/t requirements became 35 for the open eye and 125 for the closed eye conditions. With these very high Dk/t values, it is unlikely that complications will be caused by oxygen deprivation, but problems associated with inadequate tear flow and risk of bacterial infection will likely remain valid reasons to avoid extended wear under normal conditions [9]. In these cases, there are almost no other options. However, a temporary tarsorrhaphy might perform quite well in some cases. Nevertheless, as a matter of principle, contact lenses used for corneal bandages partly share the risk profile of cosmetic contact lenses, especially when the latter are overworn [10]. Microbial infections of the cornea are a major complication that arise on the basis of epithelial defects [11]. Postmarket experience reveals that infection rates are similar between silicone-hydrogel and hydrogel lenses [6, 12, 13]. Contact lenses can mask the early symptoms of the infection and thereby foster microbial spread until ophthalmological presentation. Corneal infections can ultimately induce corneal scars that interfere with visual acuity, e.g., by means of irregular astigmatism. If the scars are dense and located centrally, a keratoplasty may be the only way to improve vision in the long run. If the acute corneal infection cannot be managed by conservative means, an emergency keratoplasty is warranted to prevent florid endophthalmitis [14]. The latter is the ultimate complication from corneal infections and requires vitreoretinal surgery in most cases. Irreversible loss of visual acuity from retinal damage is not uncommon.

The benefit of bandage contact lenses therefore has to be carefully weighed against potential risks and side effects. We herein report the safety profile of bandage contact lenses in a tertiary care eye hospital in Germany on the basis of an extensive review of medical records.

## Methods

### Study design and data source

We electronically searched our outpatient electronic healthcare records information technology system for prescriptions of bandage contact lenses to analyze the safety profile of bandage lenses by investigating adverse events in electronic medical records. We did not apply any exclusion criteria.

### Bandage lens use

In our hospital, a wide variety of soft contact lens types with either conventional diameters (14.0 mm) or large diameters (17.0 up to 19.5 mm) are used. All large diameter contact lenses were made of Hydrogel material Filcon II 3 (Dk 43; H2O 75%) or Silicone-Hydrogel material Filcon V3 (Dk 60; H2O 74%) (HECHT Contactlinsen GmbH, Germany). Bandage lenses have been used almost exclusively in eyes with protracted epithelial defects from complex underlying eye conditions. Uncomplicated corneal erosions, by contrast, are commonly treated by lubrication and observation in our hospital.

Patients with intraoperatively inserted lenses were documented differently. In these instances, no conventional prescriptions were filled. As we used the prescription record to screen for this study, these patients could not be included.

Bandage lenses are normally exchanged every one to four weeks depending on several factors including the underlying disease, the condition of the ocular surface, and the state of the bandage lens itself. In Germany, the frequency of visits for patients using BCLs can vary based on the severity of their condition and the specific treatment protocol. Generally, patients are seen every 1–4 weeks for BCL replacement and monitoring. The frequency of visits was tailored to the individual needs of each patient, considering factors such as the underlying disease, the condition of the ocular surface, and the state of the BCL. For example, in cases of severe epithelial defects or persistent corneal erosions, lenses may be replaced more frequently to prevent complications such as infection or lens deposits. In contrast, for conditions with stable healing, such as postoperative care for uncomplicated surgeries, lenses may be left in place for up to four weeks. Additionally, the decision to replace a BCL may also consider patient comfort, lens displacement, and signs of reduced oxygen permeability or mechanical wear.

Topical treatments for eyes with bandage contact lenses included a combination of antibiotics and sometimes lubricants. Steroids were rarely used because they can adversely affect healing in patients with epithelial healing disorders. The choice of treatment was tailored to the underlying condition and the patient’s specific needs. A microbial examination was not performed after a routine lens change.

### Outcomes and follow-up

For each patient, we performed a longitudinal electronic search in our electronic healthcare records for the following adverse events: (1) clinically significant vision loss after bandage contact lens insertion, (2) penetrating keratoplasty after bandage contact lens insertion and (3) development of endophthalmitis after bandage contact lens insertion. It is important to note that bandage contact lenses are not used to treat endophthalmitis; rather, these cases were identified to assess potential adverse events following BCL insertion.

Clinically significant vision loss was defined as loss of at least 2 ETDRS (Early Treatment of Diabetic Retinopathy Study) lines in comparison to visual acuity at the time of bandage contact lens insertion. Penetrating keratoplasties were identified by crossmatching with the database of the Lions cornea bank Baden Wuerttemberg, which supplies our hospital with corneal grafts. Development of an endophthalmitis was assumed each time the International Classification of Diseases (ICD) code H44.0 had been entered into the administrative database of our hospital in a timely relation to insertion of the bandage contact lens.

For each of these events, we calculated the number of days since the time of inserting the bandage contact lens. Events occurring later than 100 days were not considered to be causatively related to bandage lens wear.

We manually reviewed all events occurring before this threshold of 100 days for a causal relation to bandage lens wear. For each event, we assessed whether sufficiently causal factors had already been present before bandage lens insertion or whether the event occurred in chronological correlation to the bandage lens insertion.

### Statistical analysis

All statistical analyses are descriptive in nature. No formal sample size or power calculations were performed prior to initiation of this retrospective study due to the exploratory nature of analyzing real-world data. Given the large identified sample of 638 bandage lens patients over a 10-year period at a tertiary academic medical center, this study was expected to be adequately powered to detect uncommon but clinically significant adverse event rates related to bandage lens use. However, the lack of prospective power analysis based on predefined minimally important effect sizes should be considered when interpreting results.

## Results

We identified 638 distinct patients who had been prescribed at least one bandage contact lens between 2012 and 2022. The indications and demographic data of these patients are summarized in Table 1.

**Table 1:** Baseline data of patient characteristics. Continuous variables are presented as medians [quartiles], and categorical variables are presented as counts (percentages)

Total number of patients of at least one affected eye [*n*]	638
Age at presentation [years]	62 [45/75]
Male/Female [*n*]	367 (53%)/327 (47%)
Count of bandage lenses [median/quartiles]	1 [1/2]
History of keratoplasty [*n*]	106 (15%)
Speciality service/indication of initial bandage lens prescription	
Emergency (e.g. lamellar penetration injuries, complicated corneal erosion)	589 (85%)
Cornea (e.g. neurotrophic ulcers)	55 (8%)
Neuro-ophthalmology (e.g. lagophthalmos)	14 (2%)
Glaucoma (e.g. overfiltrating blebs)	11 (2%)

We observed a total of 267 adverse events, with 147 not considered as these occurred 100 days after bandage lens prescription or later. This left 120 events for manual review, namely, 18 events of endophthalmitis, 21 penetrating keratoplasties and 81 events of clinically significant vision loss (at least 2 ETDRS lines). No specific history of exposure to water or dirt was recorded for the patients in this study. However, patients were advised to avoid such exposures as a standard precaution. All but two events were unrelated to bandage lens wear because the causative condition was already present at the time of bandage contact lens insertion or clearly of different origins, such as cataract formation. We noted two episodes of endophthalmitis from corneal ulcers that occurred in association with bandage lens insertion. These are summarized in table 2. One case was associated with limbal stem cell deficiency after an ocular burn, complicated by multiple penetrating keratoplasties and a history of herpetic ulceration. The second case involved acanthamoeba keratitis. While endophthalmitis due to acanthamoeba infection is well-documented, it is plausible that the presence of the bandage lens facilitated the infection. Thus, we included this acanthamoeba case in tables 2 to reflect the potential role of the bandage lens in exacerbating the condition.

**Table 2:** Two events of endophthalmitis with possible association to bandage contact lens wear

Age at event in years	Initial ocular condition	Delay from insertion of contact lens	Indication for bandage lens
53	Limbal stem cell deficiency after ocular burn, multiple penetrating keratoplasties, history of herpetic ulceration	30 days	Corneal erosion on graft
45	Acanthamoeba keratitis	28 days	Corneal erosion

## Discussion

Establishing the safety profile of bandage lens wear is challenging in a retrospective analysis when contact lenses are only prescribed to patients with persistent corneal defects and complex underlying conditions. These factors themselves increase the risks of keratitis and vision loss, so each adverse event must be evaluated retrospectively for a possible relation to lens use. A randomized clinical trial with a sham control group would facilitate a safety assessment by allowing direct comparison of complication rates between groups. However, such a trial would be impractical and ethically questionable. Therefore, retrospective analysis is the only way to determine the safety profile of bandage contact lenses.

To pursue this analysis, we used the time association between lens insertion and adverse events as a proxy for causation. We excluded events occurring more than 100 days after lens insertion from the safety assessments. We carefully reviewed the remaining events for evidence that the bandage lens directly caused the adverse event. We focused specifically on severe events that could be clearly identified from records: intraocular infection, visual acuity loss, and emergency keratoplasties. Mild events such as ocular discomfort or conjunctival hyperemia were not considered, as they are not clinically significant and are difficult to extract from the documentation.

After review, only two endophthalmitis events remained where bandage lens use was possibly causative, though questionable given the severe ocular comorbidities in both eyes that could independently induce endophthalmitis. It is crucial to clarify that bandage contact lenses are not used to treat endophthalmitis; these cases were observed as potential complications following BCL insertion for other underlying conditions.

Unlike cosmetic lenses, bandage lenses are often kept on the eye continuously for days. Imaginable, this is a risk factor for microbial keratitis [15].

A key reason for the good safety profile in our cohort may be that bandage lenses were not handled by the patients themselves. Lens contamination from poor hygiene is a major risk factor for keratitis from contact lens use, but this does not apply when lenses are worn uninterruptedly until removal by an ophthalmologist under optimal conditions. Close follow-up of bandage lens patients by ophthalmologists likely also prevented worsening of any keratitis.

## Conclusions

In summary, severe adverse events were rarely directly caused by bandage contact lenses with proper ophthalmologic supervision among 638 patients at a tertiary eye hospital over a 10-year period. Only two episodes of endophthalmitis could be linked to bandage lens use in our retrospective analysis.

These real-world data suggest that medically managed bandage lenses have an acceptable safety profile for supporting healing of complex corneal epithelium conditions and following ocular surgery.

Our study provides much-needed data from daily clinical practice showing low risks of vision-threatening complications attributable to bandage lenses. These findings can reassure clinicians about using bandage lenses for indicated cases with appropriate monitoring and follow-up. This information also helps support evidence-based guidelines and policy regarding bandage lens indications and best practices.

## Data Availability

No datasets were generated or analysed during the current study.

## Abbreviations

BCL: Bandage contact lenses
Dk: Oxygen permeability
ETDRS: Early Treatment of Diabetic Retinopathy Study
ICD: International Classification of Diseases
SiHy: Silicone-hydrogel
